# De novo transcriptome of the desert beetle *Microdera punctipennis* (Coleoptera: Tenebrionidae) using illumina RNA-seq technology

**DOI:** 10.1007/s11033-014-3615-6

**Published:** 2014-08-21

**Authors:** Xueying Lu, Jieqiong Li, Jianhuan Yang, Xiaoning Liu, Ji Ma

**Affiliations:** 1 Xinjiang Key Laboratory of Biological Resources and Genetic Engineering College of Life Science and Technology, Xinjiang University, 14 Shengli Road, Urumqi, 830046 China; 2 Key Laboratory of Chemistry of Plant Resources in Arid Regions, Xinjiang Technical Institute of Physics and Chemistry Chinese Academy of Sciences, Urumqi, 830011 China; 3 Department of Pediatrics, Texas Children’s Center, Dan L. Duncan Cancer Center, Baylor College of Medicine, Houston, TX USA

**Keywords:** *Microdera punctipennis*, Transcriptome, Illumina sequencing, Heat shock protein, Antifreeze protein

## Abstract

Insects in Tenebrionidae have unique stress adaptations that allow them to survive temperature extremes. We report here a gene expression profiling of *Microdera punctipennis*, a beetle in desert region, to gain a global view of its environmental adaptations. A total of 48,158,004 reads were obtained by transcriptome sequencing, and the de novo assembly yielded 56,348 unigenes with an average length of 666 bp. Based on similarity searches with a cut-off E-value of 10^−5^ against two protein sequence databases, 41,109 of the unigenes (about 72.96 %) were matched to known proteins. An in-depth analysis of the data revealed a large number of genes were associated with environmental stress, including genes that encode heat shock proteins, antifreeze proteins, and enzymes such as chitinase, trehalose, and trehalose-6-phosphate synthase. This study generated a substantial number of *M. punctipennis* transcript sequences that can be used to discover novel genes associated with stress adaptation. These sequences are a valuable resource for future studies of the desert beetle and other insects in Tenebrionidae. Transcriptome analysis based on Illumina paired-end sequencing is a powerful approach for gene discovery and molecular marker development for non-model species.

## Introduction

Deserts are among the most hostile habitats on earth. In summer, it is extremely hot during daytime, while in the depth of the winter’s night, it is surprisingly cold, as well as extremely dry in some seasons. Under these extreme conditions, small arthropods and particularly Tenebrionidae beetles are conspicuous components of the fauna. To achieve this impressive resistance to extreme stress, these small animals possess several behavioral, morphological and physiological adaptations [1–3], such as burying themselves deeply in the substrate to avoid high temperatures and extreme dry during the day [1, 4], and taking up fog-water as a water source [5–8]. Most desert tenebrionids adopt seasonal behavioral changes to avoid hostile conditions [9, 10]. Subelytral cavity, an airtight space formed by the fusion of the elytra [11, 12], is found especially in desert Tenebrionidae and it helps to lower cuticular water permeability in desert beetles [13, 14].

Desert insects have the capacity of making significant and rapid adjustments to even slight changes in environmental temperature in their physiological state, characterized by cellular desiccation, build-up of metabolic wastes and depressed metabolic activity [15]. However, even in the frozen state some complex physiological processes continue, including cryoprotectant synthesis [16, 17] and diapause development [18]. Understanding of the roles of various proteins in insect has advanced substantially in the past 20 years. The development of powerful molecular tools and the increasing ease of their application have facilitated the identification and structural characterization of novel proteins, and progress is being made on determining their function in promoting winter survival in insects. Heat shock proteins (HSPs), also known as stress proteins, play a critical role in protecting organisms from injury due to high or low temperature [19], anoxia, desiccation [20] and a range of chemical stresses [21]. Besides, it is well known that antifreeze proteins (AFPs) play important roles in protecting poikilothermic organisms from freezing by promoting supercooling and inhibiting ice formation [22]. Moreover, it is found that AFP genes also expressed in summer beetles in desert region [23, 24] and is induced by high temperature [25]. These results suggest that AFPs may play a role in the adaptation of desert insects to environment.

One of these, *Microdera punctipennis* (Coleoptera: Tenebrionidae) is an endemic beetle in the Gurbantunggut Desert in Xinjiang [26], the north west of China. It is flightless, night active; and its behavioral and morphological characteristics for desert living have been identified carefully [10]. The day-night and seasonal temperature vary greatly in this region. This extreme variation in temperature might suggest that *M. punctipennis* have evolved a range of physiological and molecular adaptations for survival. Adults of *M. punctipennis* have supercooling points below −19.6 °C, and their capacity for supercooling has been shown to increase considerably with decreasing of water in their body fluid, but the underlying molecular basis remains unknown [23]. The study of desert beetles is important because it illustrates many of the solutions evolved by arthropods to the problems engendered, in an extreme form, by life in all terrestrial environments.

RNA-Seq is a recently developed large-scale genome-wide approach that has been applied successfully to gene discovery and expression profiling, and to the study of functional, comparative and evolutionary genomics in non-model organisms for which little previous genomic information existed. RNA-Seq has the advantages of being cost effective, highly sensitive, and accurate, with a large dynamic range [27]. In the past few years, this technology has been used to investigate molecular mechanisms in insect species such as *Micrarchus* nov. sp. 2, *Tomicus yunnanensis*, and *Cryptolaemus montrouzieri* [28–30]. Here, we describe the use of Illumina/Solexa paired-end technology for de novo transcriptome analysis of *M. punctipennis*. We obtained transcriptome sequences and discovered most of the known HSP and AFP genes, as well as the genes involved in the pathways for trehalose and chitin biosynthesis. Here, for the first time, we report the genomic profile information of the arid beetle *M. punctipennis*. This study also provides an insight into the molecular pathways involved in stress adaptation in this species.

## Experimental procedures

### Insects

*M. punctipennis* beetles were collected from the southern edge of the Gurbantunggut Desert (N 44°24, E 087°51′, 444 m), Xinjiang, China. The *M. punctipennis* adults were reared at 25 °C in the laboratory. Then, the samples were frozen in liquid nitrogen and stored at −80 °C until further use.

### cDNA library generation and Illumina sequencing

Total RNA was extracted from three adult beetles using TRIzol Reagent (Sangon Biotech, China) according to the manufacturer’s instructions. The extracted RNA was assessed for quality and quantified using an Agilent 2100 Bioanalyzer (Agilent Technologies, Mississauga, Canada) with an RNA integration number (RIN) of 8, which is an algorithm for assigning integrity values to RNA measurements. For transcriptome analysis, the cDNA library was prepared using the TruSeq Sample Preparation Kit (Illumina, San Diego, CA, USA) following the manufacturer’s recommendations. Briefly, mRNA was purified from 2 μg of total RNA using oligo (dT) magnetic beads. Divalent cations were used to fragment the purified mRNA into small pieces at 94 °C for 5 min; thereby priming bias was avoided when synthesizing the cDNA. The cleaved RNA fragments were used for double-stranded cDNA synthesis using a SuperScript Double-Stranded cDNA Synthesis kit (Invitrogen, Camarillo, CA, USA) with random hexamer (N6) primers (Illumina). The synthesized cDNA was subjected to end repair and a-Tailing processes before ligation of the adaptors. The end products were purified using a 2 % TAE-agarose gel (Certified LowRange Ultra Agarose, Bio-Rad) and enriched by PCR to create the final cDNA library with sequences of approximately 300 bp. After detection using an Agilent 2100 Bioanalyzer, the cDNA library clusters were generated by cBot machine (Illumina, San Diego, CA, USA) and then sequenced in Pair-End method by Sangon Biotech (Shanghai) Co., Ltd., China using an Illumina HiSeq^TM^ 2000 (Illumina, San Diego, CA, USA) according to the manufacturer’s instructions.

### Sequence statistic and de novo assembly

Prior to assembly, the raw reads were cleaned by removing adapter sequences through the standard Illumina pipeline including the CASSAVA program (http://support.illumina.com/sequencing/sequencing_software/casava.ilmn). Low quality reads (those with quality value less than 20) and reads containing N (N represents ambiguous bases in reads), length less than 35 bp were filtered by a sliding window approach, the window size is 5 bp [31]. De novo assembly of the valid reads was performed using the November 2011 version of the Trinity program (http://trinityrnaseq.sourceforge.net/) which was designed specifically for transcriptome assembly from RNA-Seq data [32]. Briefly, Trinity combines reads of a certain length of overlap to form longer fragments and then processes them for sequence clusters with the sequence clustering software TGICL. The resultant sequences were defined as unigenes.

### Bioinformatic analysis

The assembled unigenes were searched against the NCBI nr sequence database (ftp://ncbi.nih.gov), the Swiss-Prot database (http://web.expasy.org/docs/swiss-prot_guideline.html), kyoto encyclopedia of genes and genome (KEGG, http://www.genome.jp/kegg/), cluster of orthologous groups (COG) and eukaryotic orthologous groups (KOG) (ftp://ncbi.nih.gov/pub/COG/COG) with the BLASTX algorithm (accessed in Sept 2012). The E-value cut-off was set at 10^−5^. Genes were tentatively identified based on the best hits against known sequences. Blast2GO [33] was used to predict the functions of the sequences, to assign gene ontology (GO) terms (http://www.geneontology.org/), and to predict the metabolic pathways in COG and KEGG databases. Amino acid sequences were deduced by using ORF Finder (http://www.ncbi.nlm.nih.gov/gorf/gorf.html) and GENSCAN (http://genes.mit.edu/GENSCAN.html). The putative protein sequences were used for alignment by ClustalX (v1.83) program [34]. The MEGA5.0 software [35] was used to construct the consensus phylogenetic tree by using the neighbor-joining method based on Poisson correction model. Bootstrap analysis of 1,000 replication trees was performed to evaluate the branch strength of each tree.

## Results

### Illumina high-throughput sequencing and de novo assembly

A total of 48,158,004 raw reads were obtained by HiSeq™ 2000 (Illumina) paired-end sequencing (Table 1). After a stringent filtering process 39,654,340 valid reads of average length 95 bp were obtained.

**Table 1:** Summary statistics of the sequence assembly generated from *M. punctipennis*

	Number
Number of raw reads	48,158,004
Number of valid reads (average length)	39,654,340 (95 bp)
Total unigenes (average length, N50)	56,348 (666 bp, 1,603 bp)
Number of unigenes ≥ 1,000 bp, ≥ 3,000 bp, ≥ 5,000 bp	11,568; 2,014; 287
Length range	89 bp–10,230 bp

We used the Trinity software to perform a paired end-joining de novo assembly of the valid reads. After assembly, 56,348 unigenes with an average length of 666 bp and an N50 of 1,603 bp were obtained. Of the 56,348 unigenes, 11,568 unigenes (20.52 %) were >1,000 bp long and 2,014 (3.57 %) were >3,000 bp long.

### Annotation and function assignment

**Fig. 1:**
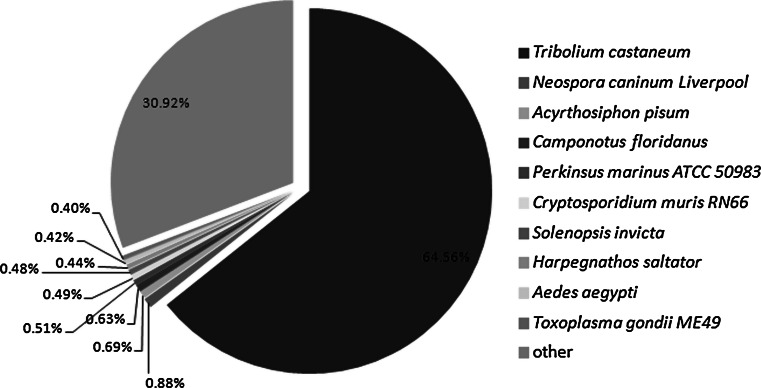
Species distribution of the BLAST hit for each unigenes. Note that nearly 64.56 % of top hits are to the beetle *T. castaneum* whose complete genome has been sequenced. We used the first hit of each sequence for analysis

To identify putative functions, the 56,348 unigenes were firstly aligned by BLASTX (E-value ≤ 10^−5^) to several protein databases: NCBI nr, UniProtKB/Swiss-Prot, UniProtKB/TrEMBL, CDD and Pfam. A total of 41,109 (72.96 %) unigenes had at least one hit to one of the databases (Table 2) and quite a large proportion (about 30 %) apparently has no significant match to any of the sequences in these databases, indicating that they may contain novel sequences and, perhaps, a high number of Coleoptera or species-specific transcripts or transcript parts (e.g. orphan UTRs). This might be expected, because there is very little sequence information from species closely related to *M. punctipennis* in these databases. The species distribution of the best match result for each sequence showed that the *M. punctipennis* sequences have 64.56 % matches with sequences from the Coleoptera species (*Tribolium castaneum*) (Fig. 1), while very low proportion (<1 %) of them have matches to other insects, for example, there was only 0.27 % (number of unigenes were 94) of them have matches to *Drosophila melanogaster* (not show independently in the figure). It demonstrated that *M. punctipennis* has a near evolution distance with *T. castaneum*.

**Table 2:** Summary statistics of functional annotation for *M. punctipennis* unigenes in public protein databases

Protein database	Number of unigene hits	Percentage
NR	35,034	62.17
SWISS-PROT	25,343	44.98
TREMBL	34,214	60.72
CDD	22,696	40.28
PFAM	19,603	34.79
Total	41,109	72.96

Pathway annotation was carried out based on the GO, COG/KOG (KOG is the eukaryotic version of COG), and KEGG databases.

#### Assignment of GO terms

**Fig. 2:**
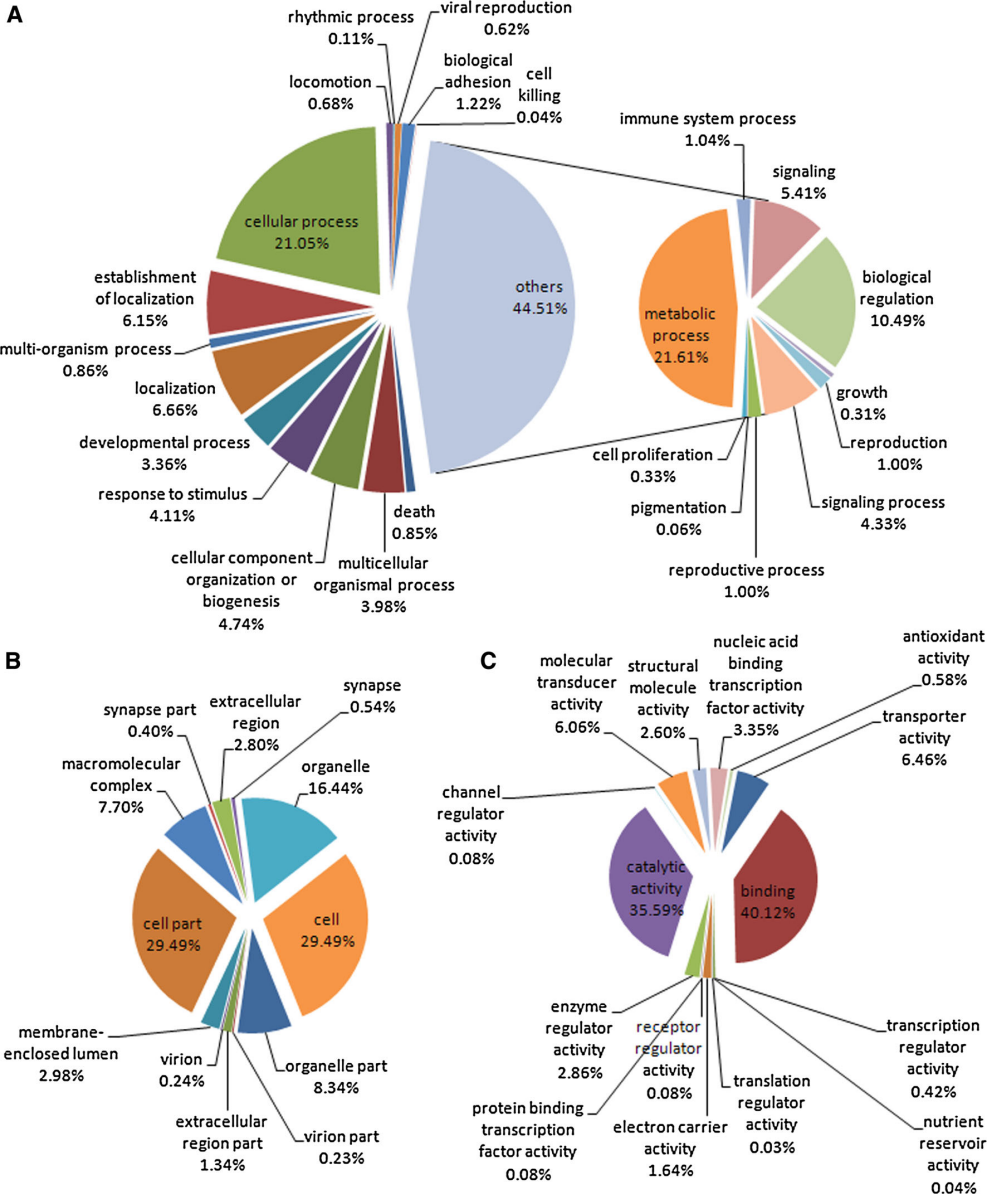
Pie charts showing gene ontology (GO) classification (level 2). GO analysis of Mp sequences corresponding to 27, 823 unigenes, as predicted for their involvement in biological processes (**a**) cellular component (**b**) and molecular function (**c**) is known

GO (http://www.geneontology.org/) is an international classification system for standardized gene functions, offering a controlled vocabulary and a strictly defined conceptualization for comprehensively describing the properties of genes and their products within any organism [36]. The three main, independent GO categories are biological processes, molecular functions, and cellular components. A total of 8,477 different GO terms were assigned to 27,823 predicted unigene-encoded peptides that previously had matches with known proteins in the UniProtKB database. The terms were from the three main GO categories and covered 52 sub-categories (functional groups) (Fig. 2). Within the biological process group, the majority of unigenes were related to metabolic process (18678, 21.16 %) and cellular process (18195, 21.05 %); within cellular component, the largest proportion were assigned to cells (19422, 29.49 %), cell part (19422, 29.42 %), and organelles (10828, 16.44 %); and within molecular function the majority were assigned as binding (20084, 40.12 %) and catalytic activity (17818, 35.59 %) including hydrolases, kinases, and transferases, allowing for the identification of genes that may be involved in secondary metabolite synthesis pathways.

#### COG/KOG classification

**Fig. 3:**
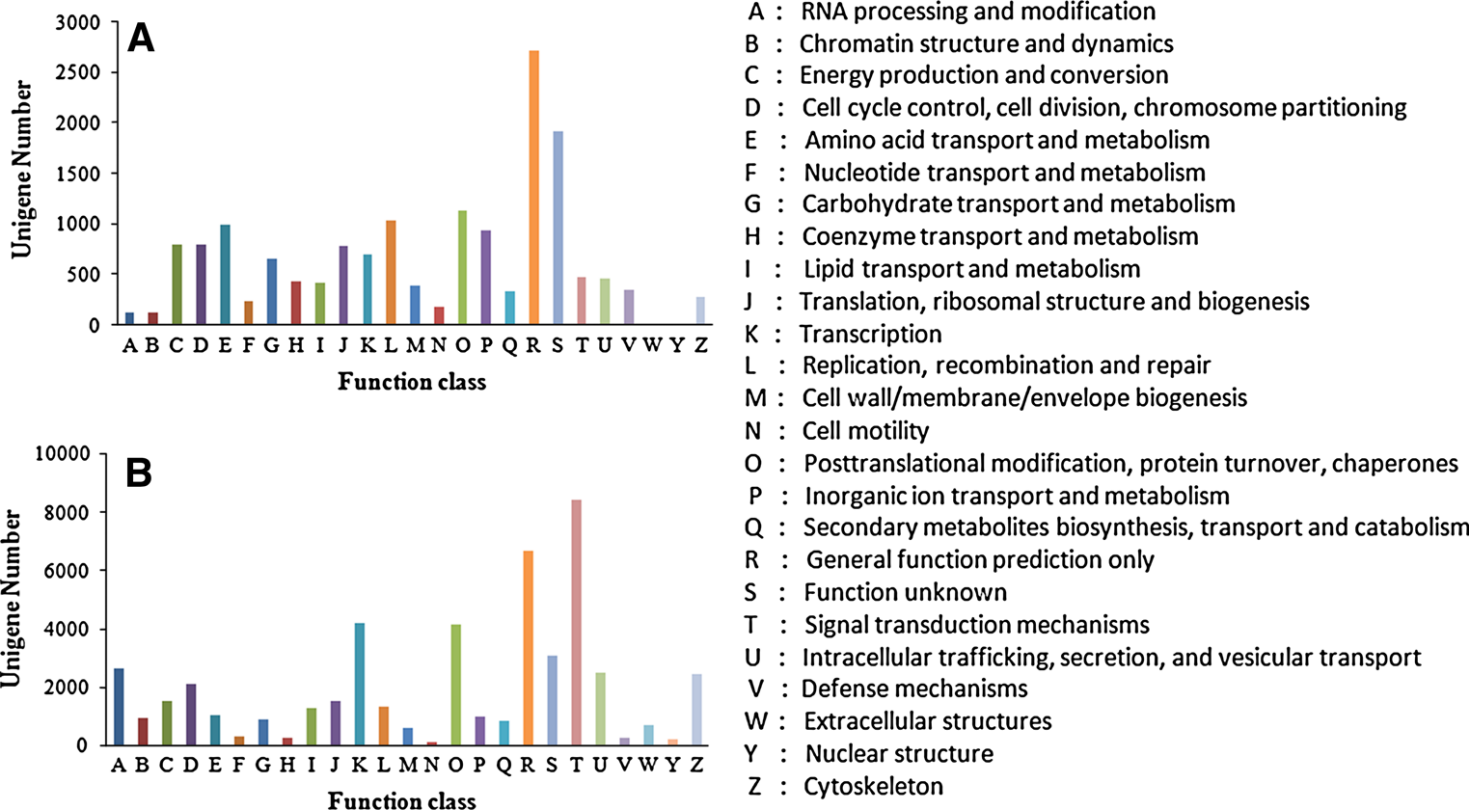
Histogram presenting clusters of orthologous group (COG/KOG) classification. **a** Of 56, 348 unigenes, 8, 980 sequences were assigned to 25 COG classification, **b** Of 56, 348 unigenes, 18, 014 sequences were assigned to 25 KOG classification

COG (http://www.ncbi.nlm.nih.gov/COG/) compares the protein sequences that are encoded in complete genomes and represents them in major phylogenetic lineages [37]. The COG construction protocol included an automatic procedure for detecting candidate sets of orthologs, manual splitting of multidomain proteins into the component domains, and subsequent manual curation and annotation [38]. Furthermore, it has been extended to complex, multicellular eukaryotes by constructing clusters of probable orthologs [39]. Altogether, 8,980 unigenes was clustered into 25 functional categories (Fig. 3a). Among of them, the ‘‘general function prediction only’’ cluster was the largest (16.82 %), followed by ‘‘function unknown’’ (11.82 %). The other larger categories were: (posttranslational modification, protein turnover, chaperones (7.01 %); replication, recombination and repair (6.39 %) amino acid transport and metabolism (6.10 %); inorganic ion transport and metabolism (5.76 %); and cell cycle control, cell division, chromosome partitioning (4.91 %). An additional 649 unigenes (4.02 %) belonged to the “carbohydrate transport and metabolism” group among which 17 unigenes were annotated as chitinase. The COG classifications shed some light on specific responses and functions of genes that may be involved in regulating various molecular processes in *M. punctipennis*. The KOG classifications corresponded to 25 of the functional categories already observed in the COG analysis (Fig. 3b).

#### Assignment of KEGG pathways

To identify the biological pathways that are active in the *M. punctipennis*, we mapped the 56,348 annotated sequences to the reference canonical pathways in KEGG. A total of 9,986 unigenes were assigned to 283 known metabolic or signaling KEGG pathways. The top 10 KEGG pathways were spliceosome (290 unigenes), purine metabolism (269), protein processing in endoplasmic reticulum (261), Huntington’s disease (239), lysosome (227), RNA transport (225), ubiquitin mediated proteolysis (221), pathways in cancer (218), endocytosis (208), and focal adhesion (204). These annotations will provide a valuable resource for investigating specific processes, functions and pathways in *M. punctipennis*.

**Fig. 4:**
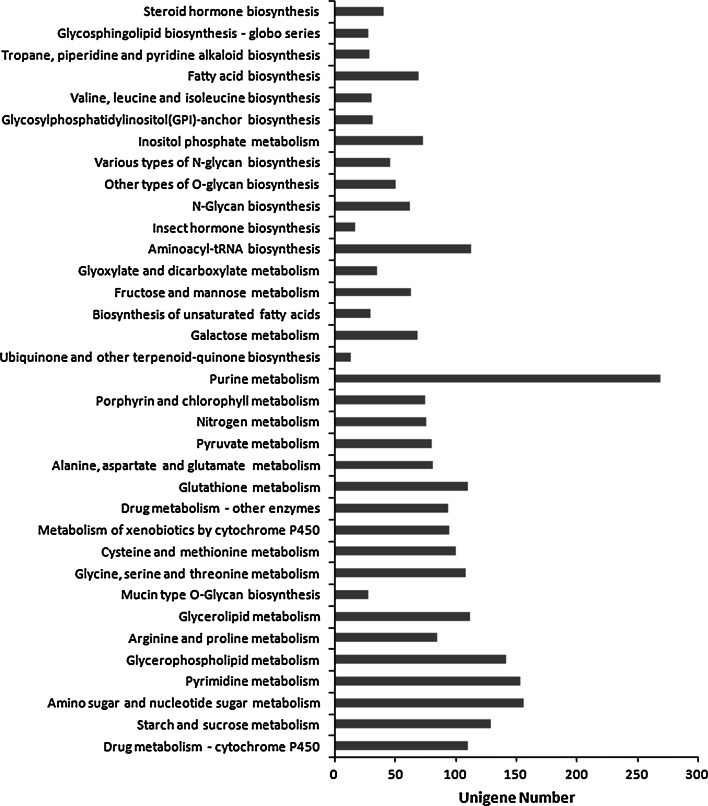
Unigenes from *M. punctipennis* related to metabolic pathways

Several of the KEGG metabolite pathways were implicated in enhancing stress defense through their generation of specific metabolites. Among the 9,986 unigenes, 1,689 were mapped to 35 pathways that are related to metabolism (Fig. 4). For example, the “purine metabolism” (ID: ko00230) and “amino sugar and nucleotide sugar metabolism” (ID: ko00520) pathways were the largest groups, containing a total of 425 unigenes among them. A further 142 and 113 unigenes were assigned to the “glycerophospholipid metabolism” (ID: ko00564) and “aminoacyl-tRNA biosynthesis” (ID: ko00970) pathways, respectively.

### Putative environment stress-related unigenes

#### Heat shock proteins

A total of 72 HSP-related unigenes were identified in the *M. punctipennis* transcriptome and 31 of them were longer than 500 bp (Table 3). The majority of the HSP-related unigenes were predicted to encode the HSP70 type. The other HSP types among the HSP-related unigenes were, HSP1, HSP9, HSP20.6, HSP90, HSP60, sHSP21, and HSP cognate 1. These results should be validated by gene cloning based on the fragments obtained here.

**Table 3:** Putatively identified HSP genes (>500 bp) in *M. punctipennis*

Gene ID	Gene Name		Length(bp)	First hit	E-value	Blast annotation/organism
Comp9597_c0_seq1	HSP70		2,391	ADB44081	6.00E−22	heat shock protein 70 [*Mantichorula semenowi*]
Comp9719_c0_seq1	HSP70		1,046	AEB52075	1.00E−161	heat shock protein 70 [*Microdera punctipennis*]
Comp9719_c0_seq2	HSP70		982	AEB52075	6.00E−162	heat shock protein 70 [*Microdera punctipennis*]
Comp9872_c0_seq1	HSP70		2,149	XP_973521	2.00E−47	PREDICTED: similar to heat shock protein 70 B2 [*Tribolium castaneum*]
Comp1983_c0_seq1	HSP70		2,054	XP_002780413	0	heat shock protein 70, putative [*Perkinsus marinus ATCC 50,983*]
Comp9719_c0_seq3	HSP70		864	ABQ39970	7.00E−120	heat shock protein 70 [*Anatolica polita borealis*]
Comp4058_c0_seq1	HSP70		818	NP_001164098	2.00E−157	heat shock protein TC005094 [*Tribolium castaneum*]
Comp2209_c0_seq1	HSP70		765	NP_001164098	1.00E−138	heat shock protein TC005094 [*Tribolium castaneum*]
Comp9464_c0_seq1	HSP70		509	AEB52075	3.00E−92	heat shock protein 70 [*Microdera punctipennis*]
Comp7346_c0_seq1	HSP70		505	AEB52075	1.00E−82	heat shock protein 70 [*Microdera punctipennis*]
Comp18449_c0_seq1	HSP70		2,054	XP_628228	0	heat shock protein, Hsp70 [*Cryptosporidium parvum Iowa II*]
Comp9719_c0_seq4	HSP70		687	ABQ39970	2.00E−112	heat shock protein 70 [*Anatolica polita borealis*]
Comp9719_c0_seq5	dnaK/70		534	ABQ39970	2.00E−77	heat shock protein 70 [*Anatolica polita borealis*]
Comp7218_c0_seq1	dnaK/70		1,816	XP_968075	4.00E−23	PREDICTED: similar to Heat shock protein cognate 1 CG8937-PA [*Tribolium castaneum*]
Comp7218_c0_seq2	dnaK/70		1,763	XP_968075	0	PREDICTED: similar to Heat shock protein cognate 1 CG8937-PA [*Tribolium castaneum*]
Comp7893_c0_seq1	dnaK/70		1,137	3LDL	7.00E−95	A Chain A, Crystal Structure Of Human Grp78
Comp69543_c0_seq1	dnaK/70		596	BAF49512	2.00E−74	heat shock protein 9 [*Branchiostoma belcheri*]
Comp4031_c0_seq1	dnaJ/70		1,043	XP_001388328	8.00E−32	heat shock protein [*Cryptosporidium parvum Iowa II*]
Comp6249_c0_seq1	CRYαB		905	XP_966780	3.00E−77	PREDICTED: similar to small heat shock protein 21 isoform 1 [*Tribolium castaneum*]
Comp10639_c0_seq1	CRYαB		897	XP_966780	1.00E−72	PREDICTED: similar to small heat shock protein 21 isoform 1 [*Tribolium castaneum*]
Comp10639_c0_seq2	CRYαB		667	XP_966780	5.00E−74	PREDICTED: similar to small heat shock protein 21 isoform 1 [*Tribolium castaneum*]
Comp5547_c0_seq1	CRYαB		682	XP_968760	6.00E−91	PREDICTED: similar to heat shock protein 1 [*Tribolium castaneum*]
Comp6543_c0_seq1	CRYαB		1,091	XP_973442	6.00E−75	PREDICTED: similar to small heat shock protein 21 [*Tribolium castaneum*]
Comp1975_c0_seq1	HSP20.6		913	XP_973685	4.00E−112	PREDICTED: similar to heat shock protein 20.6 [*Tribolium castaneum*]
Comp3391_c0_seq1		TST	719	XP_966808	3.00E−44	PREDICTED: similar to heat shock protein 67B2 [*Tribolium castaneum*]
Comp9978_c0_seq1		TST	670	XP_966808	5.00E−29	PREDICTED: similar to heat shock protein 67B2 [*Tribolium castaneum*]
Comp9978_c0_seq2		TST	653	XP_966808	6.00E−29	PREDICTED: similar to heat shock protein 67B2 [*Tribolium castaneum*]
Comp3391_c0_seq2		TST	586	XP_966808	7.00E−45	PREDICTED: similar to heat shock protein 67B2 [*Tribolium castaneum*]
Comp13432_c0_seq1		HSP90A	2,296	AAC47173	0	heat shock protein 90 [*Eimeria bovis*]
Comp9568_c0_seq1		HSPD1	2,263	XP_971630	2.00E−102	PREDICTED: similar to 60 kDa heat shock protein[*Tribolium castaneum*]
Comp11141_c0_seq3		HSP75	2,297	XP_001654758	0	heat shock protein [*Aedes aegypti*]

The annotation results for seven of the unigene sequences, Comp9719_c0_seq1, Comp9464_c0_seq1, Comp7346_c0_seq1, Comp9464_c0_seq1, Comp7346_c0_seq1, Comp113296_c0_seq1 (105 bp), and Comp9719_c0_seq6 (355 bp), are consistent with the experimental pre-clone known as *M. punctipennis* sequences in the GenBank database. The annotation results for the Comp9719_c0_seq3, Comp9719_c0_seq4, Comp9719_c0_seq5, Comp64045_c0_seq1 (124 bp) sequences are consistent with the experimental pre-clone known as sequences of *Anatolica polita boreali*.

#### Antifreeze proteins

**Fig. 5:**
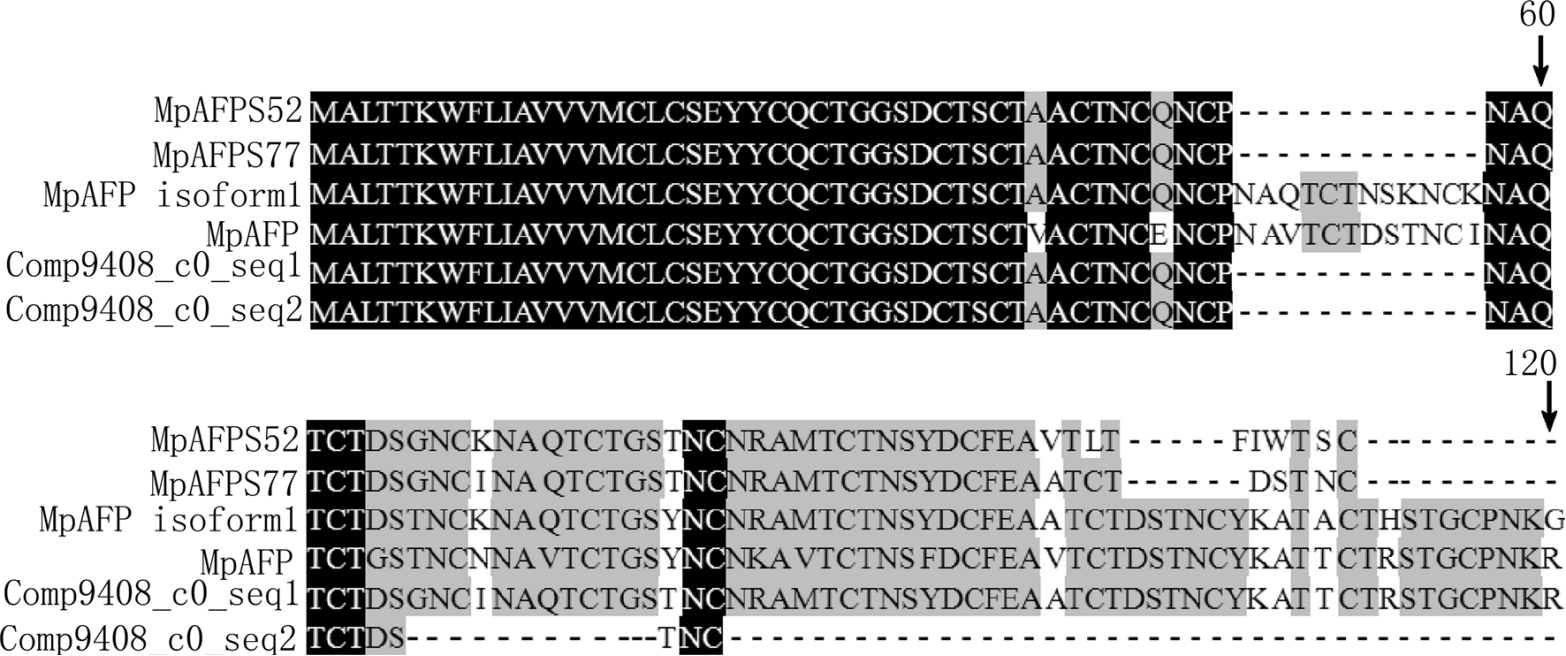
Alignment of the antifreeze protein sequences of *M. punctipennis*. Identical residues are *shaded black*, conserved substitutions are *shaded grey*. *Dash* (-) indicates insertion or deletion. The antifreeze protein name and GenBank ID of *M. punctipennies*: MpAFPS52 (ADJ93820.1), AFPS77 (ADJ93819.1), MpAFP1 (AAW67980.1), MpAFP (AAW67979.1)

**Fig. 6:**
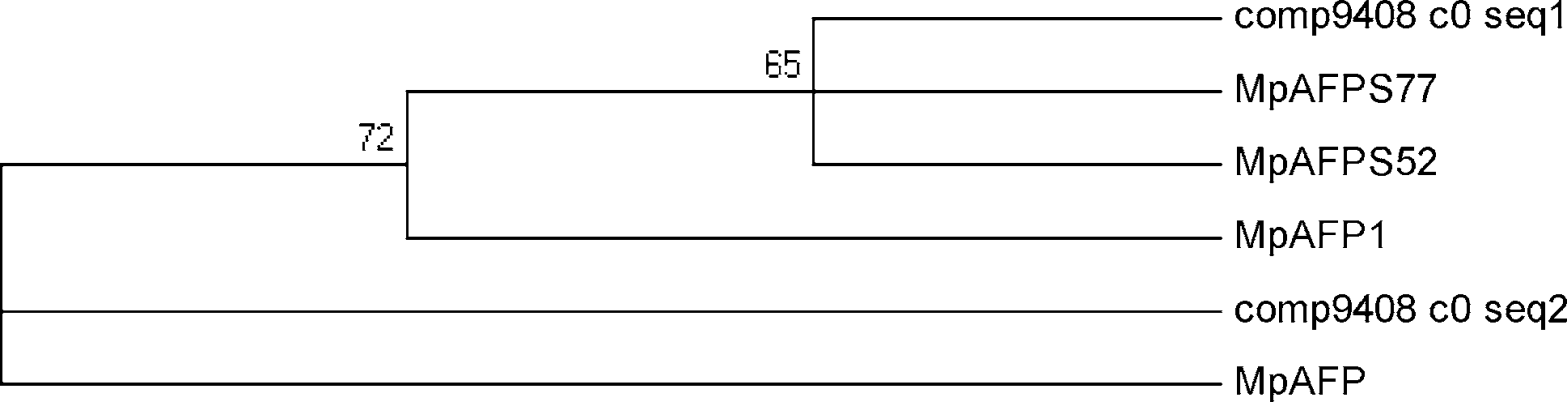
The homology relationships of *M. punctipennies* antifreeze proteins. The tree was generated using the neighbor-joining method provided by the software MEGA5 with Poisson correction for multiple amino acid substitutions, and bootstrapping test was performed with 1,000 replicates. The antifreeze protein name and GenBank ID: MpAFPS52 (ADJ93820.1), AFPS77 (ADJ93819.1), MpAFP1 (AAW67980.1), MpAFP (AAW67979.1)

Previous studies have shown that insect AFPs play important roles in cold tolerance, and there are numerous reports that the AFPs are specifically induced in insects that are exposed to low temperatures when they have been shown to improve insect freezing tolerance [40].The *M. punctipennis* anitfreeze protein (MpAFP) is Cys-, Thr-, and Ser-rich, and ExPASy prediction software indicates that its secondary structure is composed of tandem 12-residue repeats (TCTxSxxCxxAx) with extensive disulfide bond [41, 42]. Three unigenes in our assembly were identified as putatively encoding MpAFP, two of them (Comp9408_c0_seq1 and Comp9408_c0_seq2) have complete ORF. Alignment of the predicted proteins deduced from the two potentially complete unigenes showed that their percentage of identity was 78.19 % (Fig. 5), confirming the remarkable conservation within the AFP family. The relationships among the AFP sequences of *M. punctipennies* showed that Comp9408_c0_seq1 closed to MpAFPS52, MpAFPS77 and AFP1(Fig. 6). The result could provide the basis for further studies on the function of these genes.

#### Other candidates

In addition to the unigenes that have been analysed in detail above, other *M. punctipennis* unigenes with high sequence similarity to important genes related to stress metabolism and targets were identified. In particular, a number of unigenes were annotated as enzymes related to heat or cold metabolic resistance, such as trehalase, trehalose-6-phosphate synthase, chitinase, and cathepsin (Table 4). Although most of these unigenes are not full length sequences, they are nevertheless useful candidates for further characterisation by RACE to retrieve the full length cDNAs. The abundance of these transcripts demonstrates the quality of our sequencing data. This information will provide new leads for functional studies of the genes that play potential roles in beetle resistance to enviroment stress.

**Table 4:** Putative genes of interest related to stress resistance in *M. punctipennis*

Gene name	Number of unigenes had a hit with nr database
Trehalase	13
TRET1/facilitated trehalose transporter	20
Trehalose-6-phosphate synthase	5
Glycogen	23
Chitinase	65
Cathepsin	57
citrate synthase	2
ATP synthase	45
Aquaporin	10
Nucleoside diphosphate kinase	6
Cyclophilin	8
Glutathione S transferase	11
Superoxide dismutase	5

## Discussion

### Reads generation and de novo sequence assembly

The de novo assembly of short reads without a reference genome remains a challenge in spite of the development of many bioinformatics tools for data assembly and analysis [43–46]. Here, we obtained more than 4.8 billion raw reads, and assembled de novo using the Trinity software. We obtained 41,109 unigenes that matched one or more of the searched databases. The unannotated unigenes may represent novel genes whose function has not yet been identified. Specifically, the unigenes had 35,034 (62.17 %) hits to the nr database which was higher than the hits to any of the other databases (Table 2). Most of the top nr matches (first hit) were to sequences from the red flour beetle (*T*. *castaneum*) probably because: (1) it demonstrated that *M. punctipennis* has a near evolution distance with *T. castaneum*; (2) this is the only beetle with a completely sequenced genome [47]. We mapped more than 15.94 % of the *M. punctipennis* unigenes to the COG database, 31.97 % to the KOG database, 17.72 % to KEGG pathways and 49.38 % to GO terms, and found that 326 unigenes were homologous to known stress resistance genes. Many other genes and pathways related to stress adaptation were identified but need to be analyzed further.

### Heat shock protein genes

HSPs are expressed in most organisms in response to a wide range of stressful environmental conditions and are generally viewed as a protective cellular mechanism [48]. The HSP70 family includes the strictly stress-inducible HSP70 and the constitutive HSC70 (heat shock cognate proteins), the glucose-regulated protein Grp78 (BiP) [49], and the mitochondrial form mitHSP70 (grp75) [50]. In a previous study, we isolated the full length cDNA sequence of a Hsp70 gene from *M. punctipennis* (*Mphsp*70) using the RACE-PCR technique. Real-time quantitative PCR showed that the mRNA levels of *Mphsp*70 at 37 °C and 42 °C was 21.6 and 389.3 fold respectively that of the control at 25 °C, and the mRNA levels decreased as time prolonged at the high temperatures [51]. In the present transcriptome we obtained a considerable number of inducible HSPs genes (72 in total) and we speculated that these genes may help *M.* *punctipennis* adapt to the extreme desert environment. Besides, two unigenes (Comp7218_c0_seq1 and Comp7218_c0_seq2) were found similar to the sequence of HSC70 cDNA. Since HSC70 is an important part of the protein folding machinery in a cell [52, 53], the expression of HSC70 in *M.* *punctipennis* may help protect its tissues from stress.

### Antifreeze protein genes

AFPs were characterized initially in marine fishes [54, 55], where they protect their hosts from freezing by binding to and preventing the growth of seed ice crystals [56]. AFPs lower the freezing point of a solution containing ice below the melting point of the ice. AFPs function both in freeze resistance and freeze avoidance insects, thus AFPs may help insects survive most inhospitable environments.

In previous study, four isoforms of AFPs from *M. punctipennis* have been isolated and identified [25, 41, 42]. Two of the cDNAs (Mpafps77 and Mpafps52) were from beetles that were collected in summer. The deduced amino acid sequences of the MpAFPs expressed in summer are one 12-residue repeat shorter and have significantly different C-terminal end sequences compared with the MpAFPs expressed in winter [25]. Dozens of AFP isoforms have been indentified in *Choristoneura fumiferana* [57], *Tenebrio molitor* [58] and *Dendroides canadensis* [24]. The function of these AFP isoforms may be different. Six isoforms of cfAFP from *C. fumiferana* were shown development-specific expression patterns [59]. Similar to *C. fumiferana* and *T. molitor* AFPs, the MpAFPs apparently consist of many isoforms with conserved residues [60], which may play important roles in maintaining the integrity of the structure and function of the AFPs. In the present study, three unigene sequences that potentially encode AFPs were identified; their sequences were conserved when aligned with those pre-cloned (Comp9408_c0_seq1 and Comp9408_c0_seq2 *vs*. MpAFP, MpAFP1, MpAFPS52 and MpAFPS77). This sequences were obtained under different conditions, such as room temperature and cold treatment [25, 41], which suggested that different MpAFPs may have additional functions that were trigered by environmental signals.

### Metabolism related unigenes

According to the information provided by GO classification, most of unigenes in the present data were related to metabolism in the biological process. We analyzed 270 unigenes which belong to 13 different groups, and are related to metabolism of *M. punctipennis* (Table 4). These genes were grouped into the following functions: transmembrane transporter activity (GO:0022857), catalytic activity (GO:0003824), polysaccharide catabolic process (GO:0000272), cysteine-type endopeptidase activity (GO:0004197), etc. Thirteen unigenes were annotated as trehalase (a-glucoside-1-glucohydrolase, EC 3.2.1.28), which is an enzyme that hydrolyzes trehalose to yield two glucose molecules. Trehalose is the major hemolymph sugar in most insects, which acts as an indispensable substrate for energy production and macromolecular biosynthesis [61]. It is predominantly synthesized in the fat body and released into the hemolymph [62]. Trehalase plays a pivotal role in various physiological processes in insect, including flight metabolism [63], chitin synthesis during molting [64], and cold tolerance [65]. All these functions are achieved through the hydrolysis of trehalose (a-D-glucopyranosyl-a-D-glucopyranoside). In the present study, there are 20 unigenes which were annotated as facilitated trehalose transporter (TRET1), and 65 unigenes annotated as chitinase. The expression of these genes in field-collected animals reared under laboratory conditions suggests that *M. punctipennis* may control the transportation of small molecule to reduce the capacity of the intracellular osmotic potential response to stimuli under non-stress conditions.

According to the information provided by KEGG pathway assignment, 156 unigenes were related to amino sugar and nucleotide sugar metabolism (ko00520) pathway, including chitinase, GDP-mannose 4, 6 dehydratase, hexosaminidase, cytochrome-b5 reductase, UDP-glucose 4-epimerase, etc. The polyhydric alcohols (sorbitol, mannitol, ribitol, erythritol, threitol and ethylene glycol) and sugars (trehalose and glucose) are recognized as cryoprotectant [66]. The discovery of these unigenes may help us to elucidate the metabolic machanism of *M. punctipennis* that lives in desert environment.

## Conclusion

A total of 48,158,004 reads were obtained by transcriptome sequencing and the de novo assembly yielded 56,348 unigenes with an average length of 666 bp. Based on similarity searches with a cut-off E-value of 10^−5^ against two protein sequence databases, 41,109 unigenes (about 72.96 %) were matched to known proteins. The data presented in this study will contribute significantly to the rapid discovery of a wide diversity of candidate genes for this organism, and will also provide important new insights that will be useful in further studies of *M. punctipennis* genes and their functions.
